# Enhancing Omphalocele Care: Navigating Complications and Innovative Treatment Approaches

**DOI:** 10.7759/cureus.47638

**Published:** 2023-10-25

**Authors:** Ritika Malhotra, Bhavana Malhotra, Harshal Ramteke

**Affiliations:** 1 Surgery, Jawaharlal Nehru Medical College, Datta Meghe Institue of Higher Education and Research, Wardha, IND; 2 Paediatrics, Deep Chand Bandhu Hospital, New Delhi, IND

**Keywords:** treatment, complications, risk factors, embryology, giant omphalocele, gastroschisis, omphalocele, abdominal wall defect

## Abstract

Congenital abdominal wall abnormalities in infants present an interesting and difficult management problem for surgeons. Congenital malformations of the ventral abdominal wall can be diagnosed by their distinctive anatomical presentations. Among them, omphalocele, gastroschisis and umbilical cord hernias are the most frequently observed clinically detected abdominal wall anomalies. Omphalocele refers to the herniation of abdominal contents through a defect in the abdominal wall at the umbilicus with or without the presence of a sac. This article gives an insight into the embryology, risk factors, treatment, investigations and complications of omphalocele, a common congenital abdominal wall defect. There is minimal proof that environmental factors contribute to the development of omphalocele. However, there is a considerable amount of evidence which points to the importance of genetic or familial risk factors. Newborns and infants with prenatal diagnoses are the most frequently presenting patients with omphalocele to paediatric surgeons. This article describes the problems and the steps of management for handling each circumstance, as well as any further complications. Omphalocele and gastroschisis are frequently described together in many research papers. However, it's crucial to consider that they are two different conditions which vary in anatomy, pathology and associated conditions which account for the difference in their treatments and noticeably varied outcomes. Additionally, there is evidence that each has a different set of factors associated with risk for occurrence. There are no known etiologic causes that cause these abnormalities to develop. The size of the baby, the extent of the lesion, and any other disorders all affect how individuals with these congenital abdominal wall anomalies are treated.

## Introduction and background

**Figure 1:**
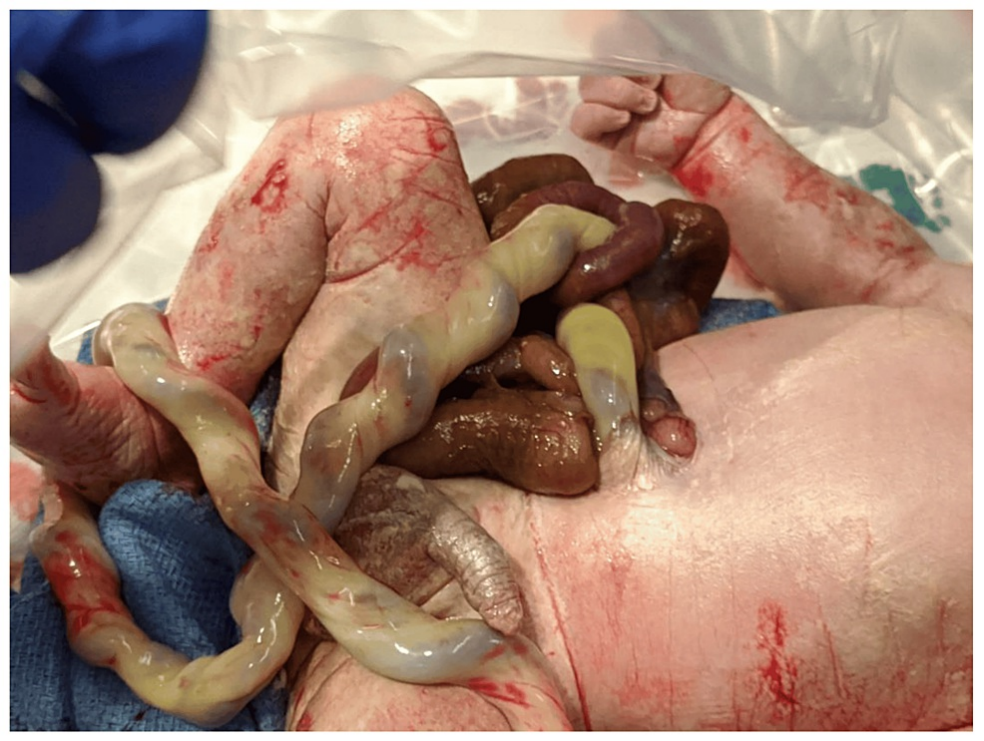
Gastroschisis Source [4]. © 2020 by the authors. Licensee MDPI, Basel, Switzerland. This article is an open access article distributed under the terms and conditions of the Creative Commons Attribution (CC BY) license (http://creativecommons.org/licenses/by/4.0/).

**Figure 2:**
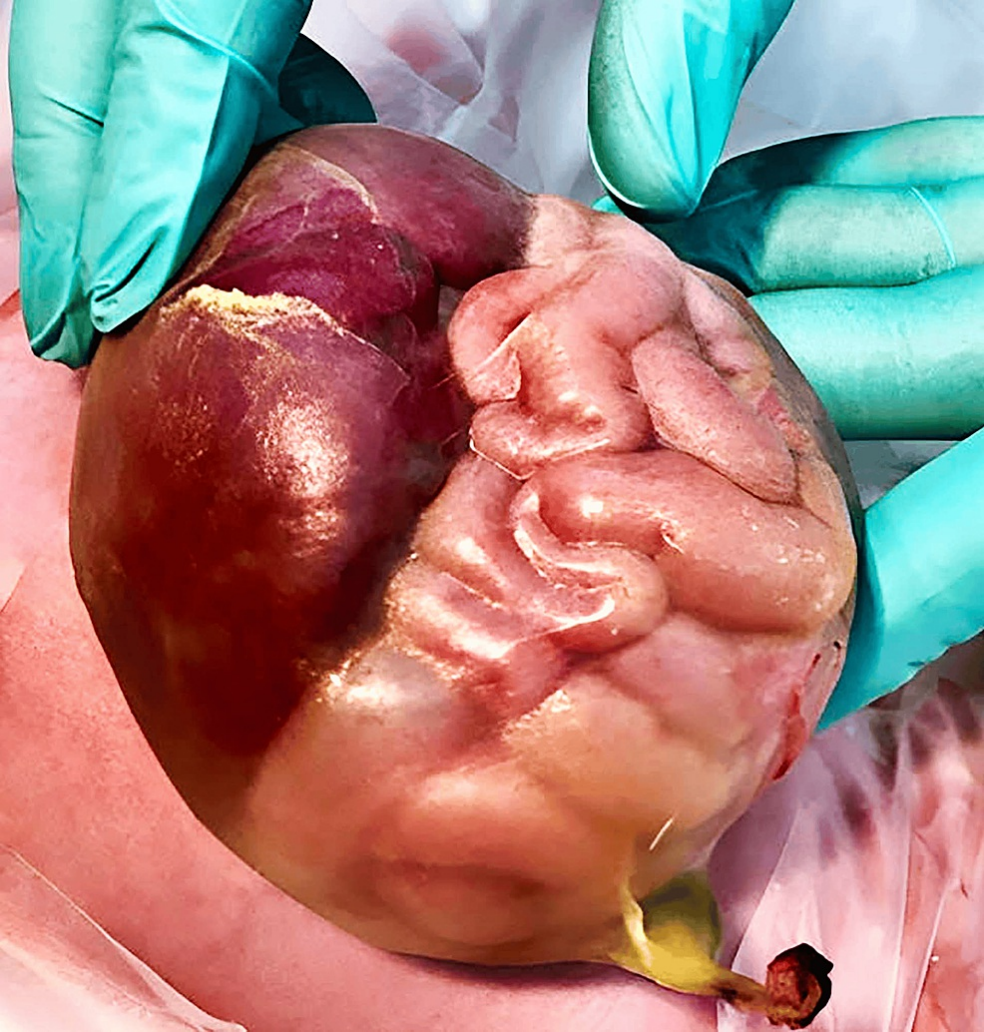
Omphalocele Source [5]. © 2021 by the authors. Licensee MDPI, Basel, Switzerland. This article is an open access article distributed under the terms and conditions of the Creative Commons Attribution (CC BY) license (http://creativecommons.org/licenses/by/4.0/).

One of the most frequently observed abdominal wall defects is omphalocele [1]. Omphalocele refers to the herniation of abdominal contents through a defect in the abdominal wall at the umbilicus, with or without the presence of a sac. This affliction affects about 1 in every 6,000 live births. Both gastroschisis and omphalocele are commonly associated with each other, as they both are congenital defects of the abdominal wall. However, their anatomy, embryo-genesis, clinical presentation and challenges differ greatly from each other [2]. An omphalocele is an abdominal wall defect of the midline. The rectus muscles are present and normal, but they protrude widely from the costal margins and do not meet at the midline. The herniated internal organs are surrounded by an intact and strong body wall, which protrudes through the umbilicus to form a membrane. The peritoneum, Wharton's jelly, and amnion make up the thin membrane that surrounds the contents of the omphalocele [3]. The differences between omphalocele and gastroschisis have been compiled in Table 1. Figure 1 and Figure 2 show gastroschisis and omphalocele, respectively.

**Table 1:** Differences between omphalocele and gastroschisis

Features	Omphalocele	Gastroschisis
Definition	Defect through the umbilicus in which the bowel fails to return inside during embryogenesis	Defect adjacent to the umbilicus
Coverings	The bowel is covered with the peritoneal sac	The bowel is not covered with peritoneum
Size of defect	Large defect; liver can also herniate	Bowel exposed
Associations	Associated with other congenital anomalies; Associated with Beckwith-Weidemann Syndrome, Trisomy 13, 18, and 21	Less congenital anomalies; Atresia and perforation following inflammation of the bowel are common

Prenatal ultrasound is a highly sensitive investigation to detect these abnormalities. They can be detected precisely during the first trimester during a nuchal scan [6]. It is common for the intestine to be herniated between six and ten weeks of gestation due to a defect in the umbilical cord. As the length of the intestine increases, it gradually retreats back into the abdomen. Furthermore, the colon rotates to the position it normally occupies during the foetus’ postpartum life. If, for reasons unknown, the intestine does not return to the abdomen before birth, the resulting condition is known as an omphalocele [7]. The rate of pregnancy termination due to these defects is extremely high as they are associated with other congenital anomalies related to the heart, kidney and bones [8]. 

## Review

Search methodology

**Figure 3:**
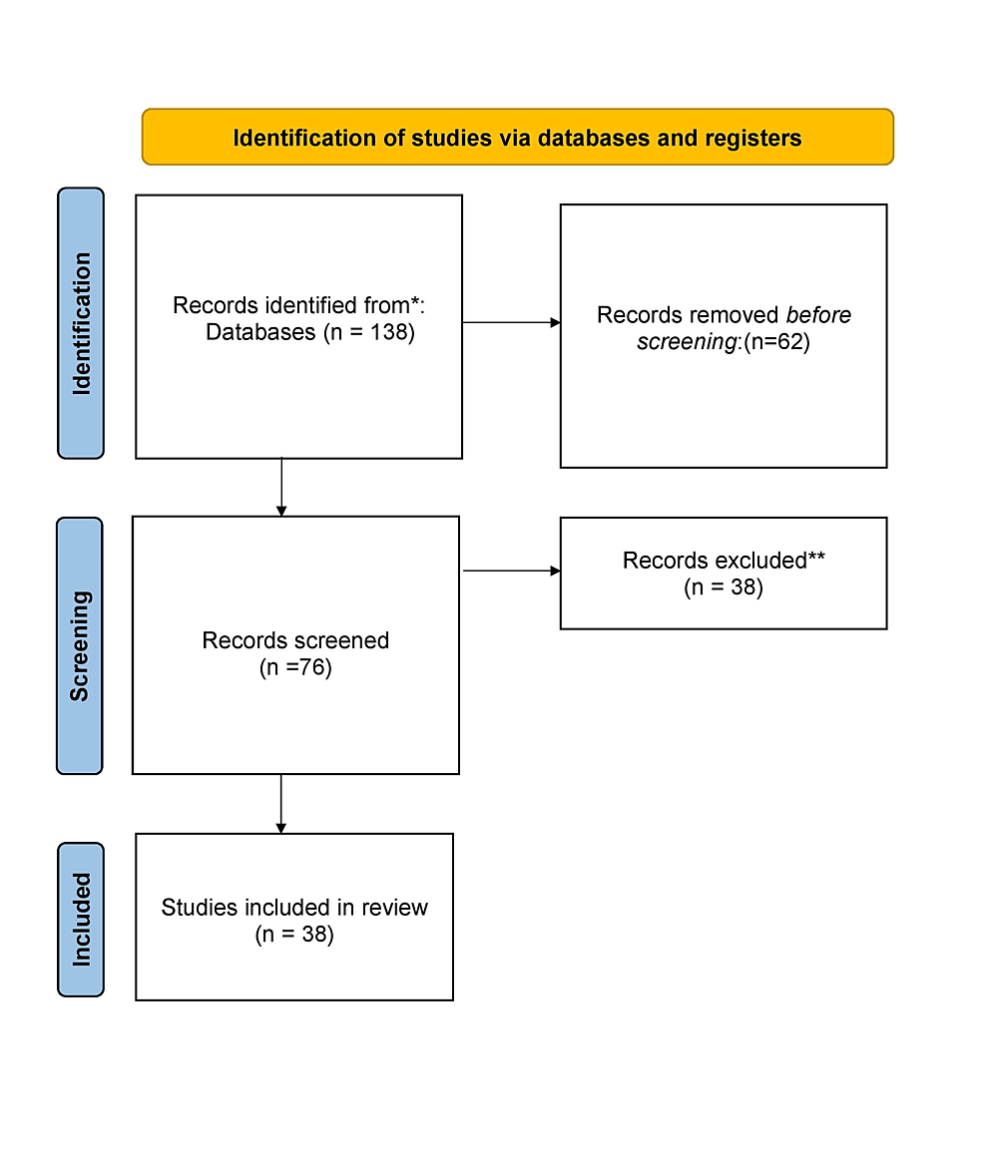
PRISMA flow diagram for search strategy

We undertook a systemic search through PubMed and Google Scholar in June 2023 using keywords such as "Omphalocele", "Gastroschisis", "Management", and "Surgery". We additionally searched for key references from bibliographies of the relevant studies. The search was updated in July 2023. Review articles, research articles and case reports published in English were explored. We searched articles from the last 7 years. In total, 38 articles were included in the review (Figure 3).

Abdominal wall growth during embryonic development

At the time of embryogenesis, the abdominal wall formation occurs through a series of events of folding and fusion of various layers. This formation can be elaborated as under. 

Formation of the Germ Layers

At the time of gastrulation, the embryo differentiates into the ectoderm, the mesoderm and the endoderm (the primary germ layers). In the end, the mesoderm differentiates into a variety of structures, including the musculoskeletal structures found in the abdominal wall.

Mesodermal Migration

Somites are a type of segmented mesodermal tissue that forms along the length of the neural tube on either side. They help create the musculoskeletal structures of the body wall. These structures include muscles and connective tissue of the abdominal wall.

Lateral Folding and Body Wall Closure

Following the lateral folding of the embryo, the midline is enclosed by the body wall. This brings together the mesoid and creates the ventral wall of the embryo. At the same time, the intestinal tube is elongated and herniated into the cord. This eventually returns, back to the abdominal wall during the later stages of development [9].

Abdominal wall closure failure in omphalocele

Omphalocele occurs when the normal closure of the abdominal wall fails, resulting in the persistent protrusion of the abdominal contents. Omphalocele occurs during the first few stages of embryonic development when the body wall does not close properly. The exact cause of this failure is multi-factorial, including genetic as well as environmental factors. Depending upon the relative size and position of the defect on the abdominal wall, various parts of the stomach and other internal organs herniate out [10]. Although the exact reason for this return of the intestine back to the abdomen failing is unknown, it is widely considered to be due to the defects in the folding of the body wall. The omphalocele may be connected to bladder or cloacal exstrophy when the folding deficit affects the caudal fold [11].

Genetic factors

The genetic factors associated with omphalocele are Trisomy 18, 13 and 21. The incidence of additional anomalies does not correlate with the extent of the abdominal wall defect in omphalocele [12].

Risk factors

The demographic factors include maternal race (African American) and advanced maternal age. High doses of vitamin E supplementation, abnormality in vitamin B12 production, maternal smoking, alcohol consumption and usage of drugs like aspirin and selective serotonin reuptake inhibitors (SSRIs) are associated with the risk factors associated with omphalocele [2].

Complications

Currently, the one-year survival rate differs between isolated omphalocele and non-isolated omphalocele. However, cases of isolated omphalocele are very rare. More than 70% of patients with isolated omphalocele have an associated chromosome or congenital abnormality. For babies with a chromosome anomaly, early mortality rates can be as high as 40% [12]. 

The most common types of chromosomal abnormalities include Beckwith-Weidemann Syndrome, Trisomy 13/Patau Syndrome, Trisomy 18/Edwards’ Syndrome and Trisomy 21/Down’s Syndrome [13,14]

Giant omphaloceles are defined as deficiencies which include more than 75% of the liver in sac and/or more than 5 cm in diameter [15]. Giant omphaloceles have been reported in many babies with poor prognosis. It is estimated that up to 35% have heart disease and 15% have a diaphragmatic hernia [16]. These babies have significantly higher neonatal complications such as respiratory distress, longer ventilator dependence, pulmonary hypoplasia and pulmonary hypertension [17,18]. These babies have a higher rate of chronic lung disease and gastroesophageal reflux disease (GERD) after the neonatal period. On the other hand, small omphaloceles have a good chance of survival as long as they do not cause fatal malformations or birth defects [19]. Omphaloceles that are smaller are more likely to have a karyotypic abnormality. In the context of other structural abnormalities, 30%-70% of cases are associated with a chromosomal abnormality or multiple malformations syndromes. The most common of these are Trisomy 13, 18 and 21, Beckwith-Wiedemann Syndrome (BWS) and Pentalogy (Cantrell) [20].

Approximately 13% of babies in their first year develop small bowel obstruction due to adhesions, and nearly 90% of these babies will require laparoscopic surgery. Some paediatricians report an adhesive bowel obstruction rate of around 15% by 10 years of age [21]. If the diagnosis and treatment is delayed, higher rates of mortality are reported. Parents should be informed about long-term vomiting, the consequences of bilious vomiting, and the urgency of surgery [22]. On the other hand, there have been rarer reports of babies with malrotations that necessitated laparotomy during childhood. A few small retrospective studies indicate a prevalence of symptomatic malrotation and volvulus in childhood of approximately 3% [23]. According to Dunn and Fonkalsrud, 40% of babies with omphalocele have an incomplete fixation of their small bowel when specifically evaluated [24].

Omphalocele can cause a wide range of respiratory conditions in newborns. These conditions can affect a baby’s health early on in life, but they can also persist into adulthood. What’s important is that the newborns born with omphalocele have an independent prognostic relationship with mortality, even after controlling factors such as gestational age and birth weight, omphalocele size, and other anomalies. Post-surgical complications such as respiratory insufficiency are commonly observed. This is due to an increase in abdominal pressure and an increase in diaphragmatic volume, resulting in a decrease in the amount of air that can be expelled [25]. Studies have shown that infants with omphalocele had decreased chest capacities, and children who survived omphalocele slowly returned to a normal chest capacity within a year or two. Reduced chest capacity and smaller lung area further caused pulmonary hypoplasia.

There is a high risk of infants developing pulmonary hypertension long with omphalocele. It is clinically diagnosed using an echocardiogram, which showed increased right ventricular systolic pressure and septal flattening. The presence of the liver in the omphalocele sac, coupled with the need for mechanical ventilation at birth, was predictive of pulmonary hypertension [26]. Omphalocele has also been associated with diaphragmatic anomalies such as the elevation of the diaphragm and ineffective diaphragmatic contractions.

To sum up, there are dozens of prenatal respiratory disorders that develop shortly after birth in patients with omphalocele. Serious respiratory impairment resulting from these disorders, particularly pulmonary hypoplasia and pulmonary hypertension, significantly increases the mortality and mortality rates of these patients. Respiratory complications can be exacerbated by surgical reduction, but most patients have a fairly rapid recovery. Additionally, other mechanical and structural disorders may affect the respiratory function [27].

Management regarding the pregnancy and delivery

Early prenatal diagnosis is important to provide parents with guidance and support throughout the pregnancy and delivery. Delivery should take place at a TEC (Tertiary Care Centre). Time and method of delivery should follow standard obstetric guidelines. Caesarean section is reserved for large omphaloceles more than 5 cm in size or for omphaloceles which involve the foetal liver. Real-time ultrasound technology allows clinicians to observe normal foetal anatomy and dynamics after 10 to 12 weeks of pregnancy. The diagnosis of omphalocele can be made by the presence of abdominal viscera at the base of the umbilical cord. With modern real-time scanners, a diagnosis can be made even at 13 weeks of gestation [20,27]. The elevation of acetylcholinesterase and alpha-fetoprotein (AFP) in the amniotic fluid with the absence of myelomeningocele is related to both omphalocele and gastroschisis [28]. There is an additional increase in the maternal serum AFP in gastroschisis as compared to omphalocele; therefore, measuring maternal serum AFP increases is a less sensitive test to diagnose omphalocele than gastroschisis. Prenatal ultrasonography performed after the first trimester may be able to detect the majority of omphalocele cases and correctly differentiate omphalocele from gastroschisis [11].

Once a diagnosis is made, genetic counselling is performed. A pedigree chart should be created to help evaluate the risk. In most cases, cell‐free DNA testing will only be used to screen for common aneuploidy disorders and should not be considered a first-tier test because of its screening nature and restrictions [29]. Omphalocele recurrence risk depends on the genetic aetiology of the omphalocele. In an isolated case, the risk of recurrence can be estimated at less than 1%. In the case of sporadic aneuploidy, the risk can be corrected after taking into account the age of the mother at the time of the diagnosis and the Trisomy [30]. Due to severe accompanying abnormalities, a foetus born with an omphalocele is at a significantly higher risk for bad outcomes. These outcomes include intrauterine growth restriction (IUGR), preterm delivery and foetal mortality. Although caesarean sections are frequently performed with big omphaloceles in order to avoid tears or dystocia during labour, there is typically no cause for early delivery [11]. The newborn might need admission to the neonatal intensive care unit (NICU) following delivery. To reduce fluid losses, the omphalocele must be quickly covered with saline-soaked gauze and an impermeable dressing [12,31,32]. Its management is done after ruling out other congenital anomalies. Pushing the bowel inside the abdomen can cause abdominal compartment syndrome. After completing airway, breathing and circulation, the abdominal wall deformity can be evaluated and addressed. The covering membrane of the omphalocele defect is checked to see if it is intact, and non-adherent dressings are used to avoid damaging the sac. Absorbable sutures can be used to try to heal a torn omphalocele sac, however, this is a temporary fix. The patient needs to be treated as a gastroschisis patient with interim covering and final reduction of the herniated material and closure of the abdominal wall if there is significant damage that cannot be repaired [11]. The patient's umbilical vessels are tied for the surgical procedure, and the whole sac is removed. The rectus sheath can be seen surrounding the defect as a result of circumferential dissection of the skin from the fascia. Signs of intestinal malposition are identified, herniated viscera is reduced, and the defect is corrected with non-absorbable sutures on the muscular and subcutaneous layers before skin closure. In other cases, one of two procedures can be performed depending on the local conditions and surgeon's preference. One of them involves the immediate closure with non-absorbable patches of synthetic mesh (gore-tex). The exterior rectus sheath is exposed for a minimum of 1.5 cm to allow for midline approximation of skin, circumferentially sutured over the fascia with non-absorbable stitches before skin closure [32]. Currently, the type of closure used is based on the size of the defect. For patients with minor defects, the primary focus is on closing the defect, and the result is completely dependent on the other anomalies involved. Due to the size of the abdominal domain, most major defects cannot be closed immediately [28]. When it comes to planning, managing, and ultimately closing the abdominal wall defect, giant omphaloceles constitute a reconstructive challenge. The purpose of reconstruction is to safely return the extra-anatomically positioned viscera to the peritoneal cavity and establish a functional abdominal wall domain [33].

A Silon chimney is another form of treatment. This is created by utilizing a silastic material that is stitched over a second row of non-absorbable threads over the fascia, evenly exposed at least 2 cm surrounding the defect. The skin is loosely stitched, and a sterile dressing is placed around it. The silo is then suspended in an incubator to provide mild and constant traction to the abdominal muscles. After a complete reduction is achieved and the abdomen is soft to palpate, a final attempt is made to close the defect, and the fascia is closed in the midline, or a prosthetic graft is made with gore-tex when closure is still not possible [34,35]. The child is reviewed periodically, where the height of the silo is reduced, and the surgeon gradually pushes the bowel into the abdomen over multiple visits. Once it reaches the level of the rectus sheath, the contents are pushed inside, and the abdomen is closed. There have also been cases where surgeons have tried using intra-abdominal tissue expanders with good outcomes. In an effort to restore a functional abdominal wall domain, tissue expansion has become a key therapy adjunct over time. Different implantation sites have been described, with the subcutaneous and intramuscular planes being the most common ones [33]. 

There are articles which cited the use of topical povidone-iodine and antibiotics as non-operative management of giant omphalocele, which showed that combination of topical povidone-iodine with powdered antibiotics promotes faster epithelialisation of the omphalocele compared to povidone-iodine alone. Additionally, the combination minimises the risk of hypothyroidism associated with povidone and iodine alone [36]. Other systemic reviews and meta-analyses have reviewed that antibiotic prophylaxis did not help to prevent the incidence of wound infections and abdominal wall hernias [37]. Mesh repair reduces the risk of recurrence by a small amount compared to suture repair for the main ventral hernia. However, an increase in seroma and surgical site infections (SSI) has been observed [38]. The kind and severity of concurrent congenital defects and underlying medical disorders largely determine the short- and long-term prognosis of children with omphalocele [11].

## Conclusions

In conclusion, omphalocele remains a complex and challenging congenital abdominal wall abnormality that demands meticulous care and management. This article has shed light on the critical aspects of omphalocele, including its definition, embryological origins, risk factors, complications, and treatment strategies. Early prenatal diagnosis through advanced imaging techniques has significantly improved our ability to identify omphalocele and provide crucial guidance to expecting parents. The management of omphalocele encompasses a range of surgical and non-operative approaches. While surgery remains the primary treatment, innovative techniques like Silon chimneys and tissue expanders have offered alternative solutions, particularly for giant omphaloceles.

It is crucial to recognise that omphalocele and gastroschisis, although both abdominal wall defects differ significantly in anatomy, aetiology, and clinical outcomes. Understanding these differences is paramount in providing tailored care to affected infants. In summary, while the challenges presented by omphalocele are significant, advances in medical science and technology have greatly improved our ability to diagnose, manage, and provide the best possible care for affected infants. Continued research and collaboration among medical professionals will further enhance our understanding and treatment of this complex condition, ultimately improving the outlook for infants born with omphalocele and gastroschisis.

## References

[1] Verla MA, Style CC, Olutoye OO (2019). Prenatal diagnosis and management of omphalocele. Semin Pediatr Surg.

[2] Frolov P, Alali J, Klein MD (2010). Clinical risk factors for gastroschisis and omphalocele in humans: a review of the literature. Pediatr Surg Int.

[3] Ledbetter DJ (2006). Gastroschisis and omphalocele. Surg Clin North Am.

[4] Bhat V, Moront M, Bhandari V (2020). Gastroschisis: a state-of-the-art review. Children (Basel).

[5] Bielicki IN, Somme S, Frongia G, Holland-Cunz SG, Vuille-Dit-Bille RN (2021). Abdominal wall defects-current treatments. Children (Basel).

[6] Prefumo F, Izzi C (2014). Fetal abdominal wall defects. Best Pract Res Clin Obstet Gynaecol.

[7] Martin LW, Torres AM (1985). Omphalocele and gastroschisis. Surg Clin North Am.

[8] Islam S (2012). Advances in surgery for abdominal wall defects: gastroschisis and omphalocele. Clin Perinatol.

[9] Sadler TW (2022). Langman’s medical embryology. https://books.google.co.in/books/about/Langman_s_Medical_Embryology.html?id=GpWkEAAAQBAJ&redir_esc=y.

[10] Sadler TW (2010). The embryologic origin of ventral body wall defects. Semin Pediatr Surg.

[11] Ledbetter DJ (2012). Congenital abdominal wall defects and reconstruction in pediatric surgery: gastroschisis and omphalocele. Surg Clin North Am.

[12] Akinkuotu AC, Sheikh F, Olutoye OO (2015). Giant omphaloceles: surgical management and perinatal outcomes. J Surg Res.

[13] Tassin M, Descriaud C, Elie C, Houfflin Debarge V, Dumez Y, Perrotin F, Benachi A (2013). Omphalocele in the first trimester: prediction of perinatal outcome. Prenat Diagn.

[14] Brantberg A, Blaas HG, Haugen SE, Eik-Nes SH (2005). Characteristics and outcome of 90 cases of fetal omphalocele. Ultrasound Obstet Gynecol.

[15] Biard JM, Wilson RD, Johnson MP (2004). Prenatally diagnosed giant omphaloceles: short- and long-term outcomes. Prenat Diagn.

[16] Raymond SL, Downard CD, St Peter SD (2019). Outcomes in omphalocele correlate with size of defect. J Pediatr Surg.

[17] Danzer E, Gerdes M, D'Agostino JA (2010). Prospective, interdisciplinary follow-up of children with prenatally diagnosed giant omphalocele: short-term neurodevelopmental outcome. J Pediatr Surg.

[18] Vachharajani AJ, Rao R, Keswani S, Mathur AM (2009). Outcomes of exomphalos: an institutional experience. Pediatr Surg Int.

[19] McNair C, Hawes J, Urquhart H (2006). Caring for the newborn with an omphalocele. Neonatal Netw.

[20] Adams AD, Stover S, Rac MW (2021). Omphalocele-what should we tell the prospective parents?. Prenat Diagn.

[21] van Eijck FC, Wijnen RM, van Goor H (2008). The incidence and morbidity of adhesions after treatment of neonates with gastroschisis and omphalocele: a 30-year review. J Pediatr Surg.

[22] Baerg JE, Munoz AN (2019). Long term complications and outcomes in omphalocele. Semin Pediatr Surg.

[23] Abdelhafeez AH, Schultz JA, Ertl A, Cassidy LD, Wagner AJ (2015). The risk of volvulus in abdominal wall defects. J Pediatr Surg.

[24] Dunn JC, Fonkalsrud EW (1997). Improved survival of infants with omphalocele. Am J Surg.

[25] Duggan E, Puligandla PS (2019). Respiratory disorders in patients with omphalocele. Semin Pediatr Surg.

[26] Hutson S, Baerg J, Deming D, St Peter SD, Hopper A, Goff DA (2017). High prevalence of pulmonary hypertension complicates the care of infants with omphalocele. Neonatology.

[27] Molenaar JC, Tibboel D (1993). Gastroschisis and omphalocele. World J Surg.

[28] Mortellaro VE, St Peter SD, Fike FB, Islam S (2011). Review of the evidence on the closure of abdominal wall defects. Pediatr Surg Int.

[29] Xu LL, Zhen L, Lou JW (2021). Can cell-free DNA testing be used in pregnancies with isolated fetal omphalocele? Preliminary evidence from cytogenetic results of prenatal cases. J Matern Fetal Neonatal Med.

[30] Warburton D, Dallaire L, Thangavelu M, Ross L, Levin B, Kline J (2004). Trisomy recurrence: a reconsideration based on North American data. Am J Hum Genet.

[31] Conner P, Vejde JH, Burgos CM (2018). Accuracy and impact of prenatal diagnosis in infants with omphalocele. Pediatr Surg Int.

[32] Roux N, Jakubowicz D, Salomon L (2018). Early surgical management for giant omphalocele: Results and prognostic factors. J Pediatr Surg.

[33] Adetayo OA, Aka AA, Ray AO (2012). The use of intra-abdominal tissue expansion for the management of giant omphaloceles: review of literature and a case report. Ann Plast Surg.

[34] Rijhwani A, Davenport M, Dawrant M, Dimitriou G, Patel S, Greenough A, Nicolaides K (2005). Definitive surgical management of antenatally diagnosed exomphalos. J Pediatr Surg.

[35] Schuster SR (1967). A new method for the staged repair of large omphaloceles. Surg Gynecol Obstet.

[36] Pandey V, Gangopadhyay AN, Gupta DK, Sharma SP, Kumar V (2014). Non-operative management of giant omphalocele with topical povidone-iodine and powdered antibiotic combination: early experience from a tertiary centre. Pediatr Surg Int.

[37] Aufenacker TJ, Koelemay MJ, Gouma DJ, Simons MP (2006). Systematic review and meta-analysis of the effectiveness of antibiotic prophylaxis in prevention of wound infection after mesh repair of abdominal wall hernia. Br J Surg.

[38] Nguyen MT, Berger RL, Hicks SC, Davila JA, Li LT, Kao LS, Liang MK (2014). Comparison of outcomes of synthetic mesh vs suture repair of elective primary ventral herniorrhaphy: a systematic review and meta-analysis. JAMA Surg.

